# The menace of saffron adulteration: Low-cost rapid identification of fake look-alike saffron using Foldscope and machine learning technology

**DOI:** 10.3389/fpls.2022.945291

**Published:** 2022-08-12

**Authors:** Amjad M. Husaini, Syed Anam Ul Haq, Asma Shabir, Amir B. Wani, Muneer A. Dedmari

**Affiliations:** ^1^Genome Engineering and Societal Biotechnology Lab, Division of Plant Biotechnology, Sher-e-Kashmir University of Agricultural Sciences and Technology of Kashmir, Srinagar, India; ^2^Technische Universität München, Munich, Germany

**Keywords:** *Crocus sativus*, Foldscope, microscopy, adulteration, fraud, machine learning, deep learning, image processing

## Abstract

Saffron authenticity is important for the saffron industry, consumers, food industry, and regulatory agencies. Herein we describe a combo of two novel methods to distinguish genuine saffron from fake in a user-friendly manner and without sophisticated instruments. A smartphone coupled with Foldscope was used to visualize characteristic features and distinguish “genuine” saffron from “fake.” Furthermore, destaining and staining agents were used to study the staining patterns. Toluidine blue staining pattern was distinct and easier to use as it stained the papillae and the margins deep purple, while its stain is lighter yellowish green toward the central axis. Further to automate the process, we tested and compared different machine learning-based classification approaches for performing the automated saffron classification into genuine or fake. We demonstrated that the deep learning-based models are efficient in learning the morphological features and classifying samples as either fake or genuine, making it much easier for end-users. This approach performed much better than conventional machine learning approaches (random forest and SVM), and the model achieved an accuracy of 99.5% and a precision of 99.3% on the test dataset. The process has increased the robustness and reliability of authenticating saffron samples. This is the first study that describes a customer-centric frugal science-based approach to creating an automated app to detect adulteration. Furthermore, a survey was conducted to assess saffron adulteration and quality. It revealed that only 40% of samples belonged to ISO Category I, while the average adulteration percentage in the remaining samples was 36.25%. After discarding the adulterants from crude samples, their quality parameters improved significantly, elevating these from ISO category III to Category II. Conversely, it also means that Categories II and III saffron are more prone to and favored for adulteration by fraudsters.

## Introduction

Saffron (*Crocus sativus* L.) is a highly remunerative cash crop and a source of luxury spice obtained from handpicked flowers as dried crimson stigmas (Kafi et al., 2018). According to Food and Agricultural Organization (FAO), it forms “a loosely matted mass of dark, reddish-brown flattened threads, among which a few narrower yellow ones can be distinguished. The upper, enlarged part of the flattened threads is the stigma of the flower, the lower narrower portion is the style” (Husaini et al., 2010a). Saffron bioactive compounds have immense therapeutic properties useful for coronary artery diseases, neurodegenerative disorders, bronchitis, asthma, diabetes, fever, and colds. It has the potential to help tackle problems associated with severe acute respiratory syndrome (COVID-19) patients and post-COVID-19 problems (Ahmed and Husaini, 2021). It can help manage stress and anxiety during isolation, quarantine, and lockdowns (Husaini et al., 2021). Owing to all these beneficial properties and as an immunity booster, saffron extracts may be added to some drug formulations in future (Husaini et al., 2022). These properties and their importance in religious rituals of many communities make it costly and hence prone to adulterations. Some have even advocated its cultivation in kitchen gardens to ensure the supply of pure saffron for household use (Husaini and Wani, 2020).

The best quality saffron is usually sold in filaments (Melnyk et al., 2010; Nehvi and Yasmin, 2021); therefore, in the present study, we focused on filamentous saffron. Different kinds of fake products sold under the name of “saffron” are reported in the literature (Husaini et al., 2010b; Heidarbeigi et al., 2015). The most common fraudulent practice includes artificial dyeing of some selected plant materials, making these look similar to saffron. According to a study on saffron sold in India, only 52% are genuine, 30% are poor grade, and 17% are adulterated (Husaini et al., 2010a). This menace of saffron adulteration is mushrooming as a white-collar fraud at a tremendous pace (Husaini et al., 2010a, 2013; Er et al., 2017).

According to the ISO 3632 standards (ISO, 2010, 2011), up to 1% (w/w) of foreign material is permitted in third-class products. Several chromatographic and spectroscopic methods are used for detecting saffron adulterants (Alonso et al., 1998; Lozano et al., 1999; Haghighi et al., 2007; Sabatino et al., 2011; Er et al., 2017). Moreover, several biotechnological and molecular methods are also employed to detect plant adulterants in saffron (Ma et al., 2001; Javanmardi et al., 2011; Marieschi et al., 2012; Babaei et al., 2014; Torelli et al., 2014; Petrakis et al., 2015; Kumari et al., 2021). These methods are useful for detecting low amounts (up to 1%) of bulking materials and are ideal for checking the purity of the product. However, all these analytical methods are too complicated, expensive, and need sophisticated instrumentation and higher skill levels of experts. The lack of sophisticated laboratories and ineffective law enforcement adds to the constraints in handling cheating by retailers. Some affordable methods like simple microscopy or spectrometry have their limitations too. While a conventional microscope is a fairly expensive instrument seldom used by common people, UV-vis spectrophotometric method used in labs does not detect saffron contamination up to 20% (w/w) (Sabatino et al., 2011). None of the methods developed so far is easily accessible to customers or retailers. Hence, there is a need to invent faster, low-cost screening methods for detecting saffron adulteration and fictitious look-alike versions of saffron, and make these easily accessible to the end-users.

There is a need to have a customer-centric rather than a lab-centric approach. We want to change the standpoint of looking at the problem by bringing the customer directly into the screening procedure. Customers should be able to check the authenticity of a particular sample on a retail scale because saffron being expensive, is generally sold in small packings of 1–5 grams. The present study focuses on “fictitious look-alike” versions of saffron sold in the markets under the names of fictitious brands on a commercial scale around religious places, spice markets, individual retailers, and the unorganized sector. We aim to share a customer-friendly technology that is the first of its kind and does not depend on sophisticated instrumentation.

Recent developments in “frugal science” have made a monocular origami-based low-cost optical microscope, called Foldscope, commercially available and easily accessible (Cybulski et al., 2014; Moreno-Roman and Bobick, 2022). Similarly, there are tremendous advances in artificial intelligence-based solutions and machine learning (Ben Ayed and Hanana, 2021; Janiesch et al., 2021; Vijayakumar and Balakrishnan, 2021; Greener et al., 2022). We explored these developments and developed two methods for the self-detection of fake saffron by customers and retailers. One method uses Foldscope in combination with chemical staining and destaining technique for developing a printed poster to detect fake look-alike saffron through visual comparison. The effect of different dyes on the staining pattern of the samples was studied so that even school children could use this technique. The second method uses deep learning for image classification to automatically identify genuine from fake look-alike saffron samples. The method uses Foldscope and a mobile application (app) to automate the process without using any invasive procedure. It is time-efficient and can be used by people who do not have much knowledge about the domain (Saffron). To the best of our knowledge, this is the first time that Foldscope and machine learning have been used to authenticate saffron as fake/genuine and provide user-friendly testing access to a broader audience.

## Materials and methods

### Survey and collection of samples for analysis

The collection of plant material and all experiments were performed following relevant institutional, national, and international guidelines and legislation. Direct interactions were done with saffron farmers, traders, and consumers/tourists to know their experiences and to find out the nature of adulterants being commonly used by fraudsters. Only a few cooperated in giving some basic information about adulteration methods.

#### Categorization of genuine and fake look-alike samples

**Figure 1:**
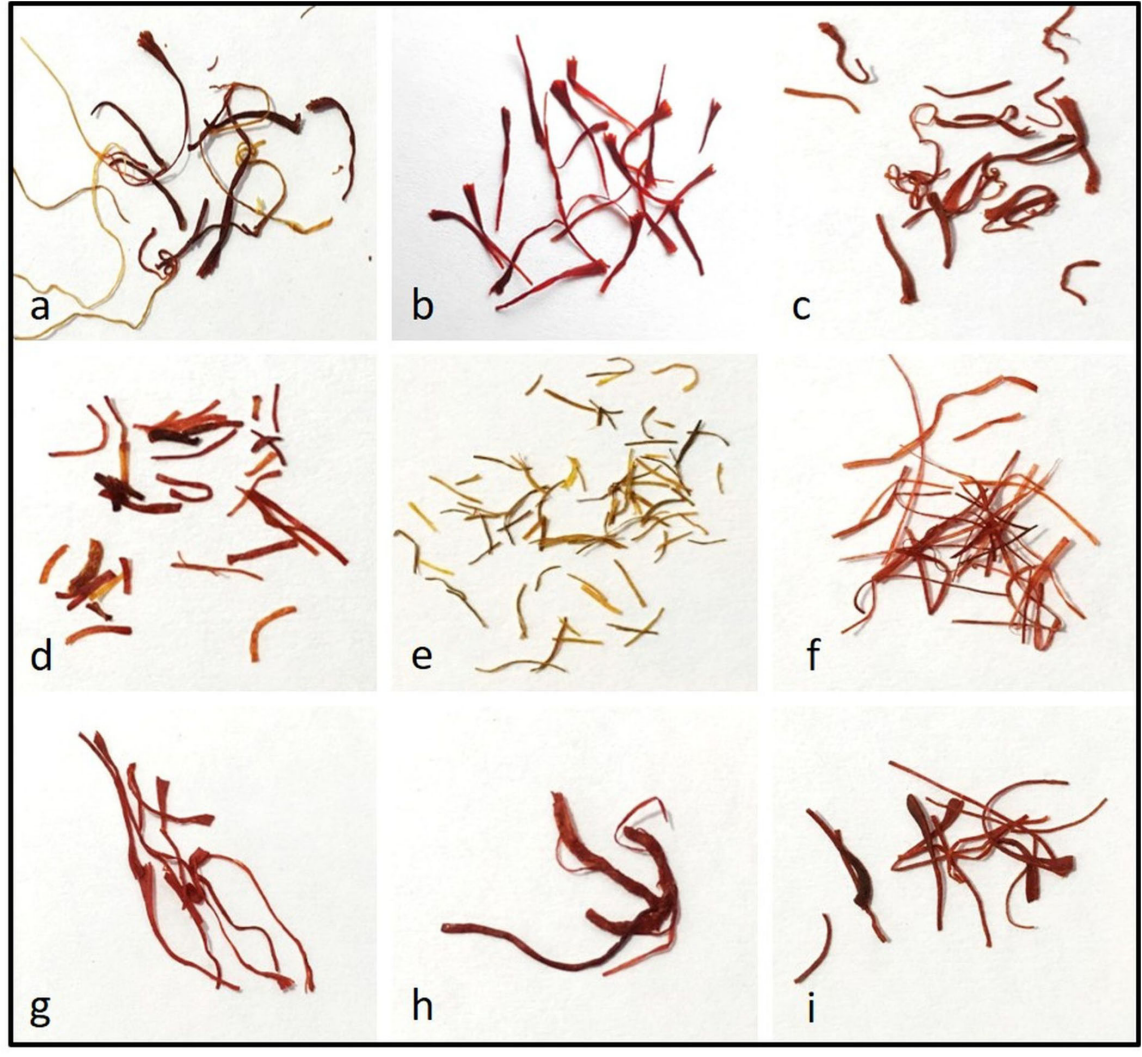
Photographs of closely resembling samples as visualized by a naked eye: Genuine saffron **(a)** saffron in filaments (Laccha), **(b)** saffron in cut filaments (Mongra); fake samples, **(c)** unknown look-alike, **(d)** unknown look-alike, **(e)** neon yellow dyed corn silk, **(f)** maroon red dyed corn silk, **(g)** unknown look-alike, **(h)** saffron stamen, and **(i)** paper strips.

A total of nine diverse classes of samples were used in the present study (Table 1). Seven classes belonged to the fictitious look-alike saffron filaments collected from open markets in India, while two classes belonged to genuine saffron grades (known as “Laccha” in native Kashmiri [“saffron in filaments” as per ISO3632 and IS5453] and “Mongra” [saffron processed using a technique indigenous to Kashmir or “saffron in cut filaments” as per ISO3632 and IS5453]) (Figure 1). “Laccha” is the vernacular Kashmiri term for saffron filament with style and “Mongra” for a locally processed grade of cut filaments lacking style.

**Table 1:** Distinctive morphological features in saffron and look-alikes as visualized using Foldscope.

**Sample Class and description**	**Characteristic features**
Class 1: “Saffron in filaments” (ISO3632)/Laccha (IS5453)	Serrated distal trumpet shaped top with distinct papillae; striated texture dotted with pit like structures; presence/absence of large pollen grains.
Class 2: “Saffron in cut filaments” (ISO3632)/Saffron processed using a technique indigenous to Kashmir “Mongra” (IS5453)	Serrated distal trumpet shaped top with distinct papillae; presence/absence of large pollen grains.
Class 3: Fake-1 “Unknown look-alike”	Serrated top exhibiting typical trumpet shaped structure; epidermal papillose protuberances absent; pollen like granules present. It may be stigma of another flower.
Class 4: Fake-2 “Unknown look-alike”	Serrations as well as other distinguishing features of authentic saffron absent. Long flattened structure with smooth margins and even width.
Class 5: Fake-3 “Corn silk dyed maroon”	Resembles stigma lobes; Smooth margins with no distal serrations or papillose protuberances. Long flattened structure with even width.
Class 6: Fake-4 “Corn silk dyed neon yellow”	Resembles style; Serrations as well as the papillose protuberances absent; Long flat structure with smooth margins and even width; pollen grains absent.
Class 7: Fake-5 “Unknown look-alike”	Wide and serrated top margins with narrow stalk resembling funnel. Papillose protuberances and texture distinctive of saffron stigma absent; Pollen grains absent. The sample structure disintegrates quickly in solvents and reveals a single thin, long thread like fiber.
Class 8: Fake-6 “Dyed saffron stamen”	Top margin wide and trumpet shaped; Large number of pollen grains present along the stalk and top regions; Papillose protuberances absent.
Class 9: Fake-7 “Dyed paper strips”	Smooth and flat, funnel shaped wide top margin with a narrow stalk; no serrations; It disintegrates quickly in solvents and reveals a single thin, long thread like fiber.

### Microscopic study

Samples were observed under stereo-microscope (Olympus SZX16 using software LCmicro-2016-17 version) and Foldscope. Foldscope is an origami-based optical microscope developed at the University of Stanford, USA and designed to cost <US$1. It weighs about 8–10 g and provides a magnification of 140×. It does not require external power and can survive being dropped from a three-story building (Cybulski et al., 2014; Joshi and Bhosale, 2018). With good resolution cell phone cameras, direct imaging is possible. Alternatively, the image can be viewed on a frosted sheet (thin velum) which can be placed above the lens.

To develop the Foldscope-based method, we used 2,250 filaments belonging to nine classes, with at least 250 individual filaments from each class for microscopic study. Single strands of dry, intact filaments were placed directly on clean, dry glass slides and covered with transparent cello-tape (Supplementary Figure S1f). These were observed under Foldscope in natural sunlight. Images were obtained by coupling the Foldscope with a cell phone (iPhone SE) using a custom magnetic coupler. The Foldscope magnification is 140×, which was further enhanced digitally by the zooming function of the smartphone having a 12-megapixel resolution camera. Observations were recorded for morphological features like (a) papillose protuberances; (b) margins; (c) serrations; (d) texture; (e) dyeing patterns; (f) pubescence; (g) pollen grains.

### De-pigmentation

In order to distinguish between artificially dyed samples and genuine ones, the dry filaments were de-pigmented by suspending in methanol (100%) for 4 h, followed by washing with 1:1 (methanol: water) 3–4 times.

### Staining

For staining, dry filaments were placed in a 1% staining solution of each staining agent (toluidine blue, safranin O, iodine, fast green, crystal violet) for 1–2 min. Staining was followed by washing with water to remove excess stain. Filaments were placed on clean glass slides. Filaments were covered with coverslip after putting a drop of water on them and observed under Foldscope. Photographs were taken with a smartphone.

### Machine learning

Before using machine learning, we tried a simpler image processing technique. As genuine saffron sample images have papillae on stigma, while being absent in fake saffron samples, we converted all images to single channel image, i.e., gray image, and employed the Canny edge detector multi-stage image processing algorithm to detect the edges in the image. We counted the number of contours on the edges in the image based on the highest gradient difference. Subsequently we used neural networks to fine tune the process.

Neural networks (NNs) are a subset of ML and basic components of the deep learning algorithm. Convolutional neural network (CNN) is a special form of NN that performs better with high-dimensional data like images and videos, and it allows faster training and reduces model complexity. We used deep neural network architecture based on ResNet18 (He et al., 2016) and Densenet121 (Huang et al., 2017) networks, with different model parameters and states. We used Python version 3.7.13 as a programming language, OpenCV version 4.1.2 for image processing, fastai version 1.0.61, backed by PyTorch version 1.11.0, as a deep learning framework, and scikit-learn version 1.0.2 as a machine learning library. We used NVIDIA Tesla K80 GPU with 12 GBs of memory capacity and Intel Xeon 2.20 GHz CPU with 12 GBs of memory capacity. We modified the last layer of networks and used it as a binary classifier with two labels, separating genuine from fake saffron samples.

### Baseline and comparative methods

To ablatively test the introduction of ML algorithm for the classification of fake and genuine saffron samples, we compared NN with the conventional ML algorithms, i.e., random forestand SVM. Evaluation of the different NNs and their variants was also done. For training the random forest model, we used 100 trees in the forest and two as the minimum number of splits for the internal node. The maximum depth of the tree is expanded until all leaves contain less than the minimum number of split samples. Gini impurity was employed to measure the quality of a split. SVM model was trained using radial basis function (RBF) kernel. During the training process, model leveraged squared hinge loss for the optimization, with 0.001 as the tolerance for the stopping criteria.

Three experiments per network were conducted for deep neural networks, namely ResNet18 and DenseNet121. First, the model weights were randomly initialized using the Kaiming initialization (He et al., 2015) approach. Second, convolutional layers of the deep neural network were initialized with pre-trained ImageNet (Deng et al., 2009) weights. The layers were kept in a frozen state during training, while the last two layers were initialized with random weights and updated as training proceeded. And finally, all the layers of both the networks with pre-trained weights were unfrozen, and during training, all the weights belonging to the layers got updated based on the loss function optimization.

Random lightning and contrast changes were performed to further enhance the training and generalize the process. Real-time data augmentation of random flip with 50% probability was also applied to training samples. These augmentations help overcome the overfitting of the model on training data and improve the overall model accuracy. Models were trained using an Adam optimizer with a learning rate of 0.001 and momentum of 0.9. Binary cross entropy was used as a loss function due to the binary classification nature of the task, i.e., a sample can be either fake or genuine. All the variations of both neural networks were trained for 15 epochs.

### Data acquisition and pre-processing

During the data acquisition phase, we randomly captured multiple images of the same filament for different samples to incorporate the variation that might arise due to clicking the image *via* smartphone from different angles, orientation, contrast, etc. It helps to make the dataset more generalized, to avoid neglecting the possible scenarios in the real-world, where users can click images with uncertainty toward any assumption, which in turn resulted in 3,794 images in total; out of these, 1,434 images belong to genuine samples and 2,360 images to fake samples. The dataset consisted of dried, with, and without stained saffron sample images to include the tolerance toward different processing performed on saffron strands.

In the pre-processing step, each image was resized to 224 × 244 × 3 (width × height × color channels) dimension to decrease the computational load and create a uniformly sized dataset. After resizing, the pixel values of images were subtracted by the mean and divided by the standard deviation. This process brings all image pixel values between 0 and 1 range and allows faster convergence later in the model training.

For the experimentation purpose, the saffron dataset was split into a training dataset containing ~72% (2,732 samples), a validation dataset containing ~18% (683 samples), and a test dataset consisting of ~10% (379 samples). The training dataset was used to train the above-mentioned models in a supervised learning fashion, where input was a saffron sample image and the label was the class it belongs to, i.e., fake or genuine. The validation dataset was handy to validate and select the best-trained model in an unbiased manner while finetuning the model hyperparameters. Last but not least, since the test dataset was not used during the training phase, it depicts the real-world behavior and allows for the evaluation of the final model.

### Machine learning algorithms for classification

Machine learning algorithms are mainly categorized into unsupervised, supervised, and reinforcement learning. The classification task falls under the supervised learning algorithm, where training takes place based on the pre-labeled data. During training, the algorithm learns the pattern from the labeled data (Veronese et al., 2013). Once trained, the algorithm assigns a new label to the new and unseen data and classifies the sample. There are multiple ML-based classification algorithms available, and, in this work, we explored random forest (Verma and Achutha, 2016), support vector machine (SVM) (Le et al., 2012), and multiple variations of CNN.

Random forest is an ensemble learning method and consists of a number of decision trees. Each decision tree predicts a class associated with the data sample, and the class with the maximum number of votes is taken as a final prediction. This combined approach adds robustness toward errors linked with the individual decision tree predictions.

SVM algorithm takes data samples during training as input and tries to find the optimal hyperplane in an N-dimensional space, where N is the number of input features. This hyperplane is a decision boundary and distinguishes the data samples into different classes.

#### Performance evaluation

For measuring the performance of all the trained ML-based classification models, accuracy and precision were employed. The classification accuracy was calculated as:


(1) $$\begin{array}{r} accuracy=\frac{\left(TP+TN\right)}{\left(TP+FP+TN+FN\right)} \end{array}$$

True positive (TP) represents the saffron samples correctly classified as genuine samples in the above equation. False positive (FP) represents the saffron samples falsely classified as genuine samples. True negative (TN) represents the saffron samples correctly classified as fake samples, while false negative (FP) represents the genuine saffron samples falsely classified as fake.

For the evaluation, accuracy focuses on the fraction of the classification of samples, both fake and genuine, corrected as predicted by the model.

The classification precision is represented as:


(2) $$\begin{array}{r} precision=\frac{TP}{TP+FP} \end{array}$$

Precision performance metric quantifies the number of correctly classified genuine saffron samples by the trained model.

#### Assessment of adulteration and quality estimation

Eighty crude saffron samples were collected from eight cities/towns of Jammu and Kashmir, India. Ten samples were collected from each city/town, with two packets of 1 g each bought from each vendor. All the 80 samples were then screened for adulteration using Foldscope. The quantum of adulteration in each sample was determined and expressed as adulteration percentage, and then averaged for each location. Spectrophotometer-based quality of the crude and the pure samples was determined to categorize these into Categories I, II, and III as per the ISO 3632.

## Results and discussion

### Limitations of saffron quality and adulteration detection methods

The quality and the commercial value of saffron are based on an estimation of coloring power, bitter taste, and aroma (Carmona et al., 2007; Kafi et al., 2018). It is certified in the international trade market following the International Organization for Standardization (ISO) 3632 Normative (Husaini et al., 2010b). Regardless of the fraudulent practice, it is challenging to identify commercial frauds in saffron because changes in physical, chemical, and organoleptic characteristics are not easily identifiable (Koocheki and Milani, 2020). Artificial intelligence technique-based artificial neural network and electronic nose have been used for quality control of saffron using its aroma fingerprint and distinguishing it from the samples mixed with safflower or corn stigma up to a proportion of 50% (w/w) (Heidarbeigi et al., 2015). The technique can detect adulterated saffron with a percentage classification accuracy of 86.87%.

An electronic nose is used to determine the geographic origins of saffron with 90% of confidence (Carmona et al., 2006). The principle of this detection is based on the differences in dehydration techniques followed in different countries and the consequent changes in the composition of volatile compounds of saffron. Several fake and original products like sunflower oil, corn oil, sesame oil, tea, and coffee have also been detected using the electronic nose (Hai and Wang, 2006; Mildner-Szkudlarz and Jeleń, 2008; Son et al., 2009). The most significant limitation with the users of such electronic sensors (e-nose and e-tongue) is the requirement of strictly controlling sample preparation, sampling, and data processing. At the same time, training a sensory panel is time-consuming and expensive. Moreover, these sensors are very sensitive to temperature, humidity, pressure, gas velocity, and vapor concentration (Tan and Xu, 2020).

Several studies have combined many techniques and used multiple types of sensors through the fusion technique to overcome the above-discussed limitations, but with limited success (Kiani et al., 2018). Contrary to the previously discussed methods, our aim is not to develop a method for detecting adulterants or extraneous “powdery material” in the saffron “powder” or the mixing of “different grades” of genuine saffron. Our paper focuses on the “filamentous” adulterants and fictitious “look-alike” versions of saffron commercially sold in markets.

### Stigma papillae are the characteristic morphological markers of genuine saffron

The stigma of *C. sativus* consists of three orange-red trumpet-shaped lobes, and it is papillate on the rim, and the average length is 3 cm. *C. sativus* pollen tube growth in intra- and interspecific pollinations has been studied in detail under a microscope (Chichiriccò, 1984; Chichiricco and Caiola, 1986). The stigma surface of saffron is of the dry type, as in the case of many other *Crocus* species (Heslop-Harrison and Heslop-Harrison, 1975; Heslop-Harrison, 1977; Caiola and Chichiriccò, 1991). While the stigmas of *Crocus sativus* and its allies *C. cartwrightianus, C. thomasii*, and *C. hadriaticus* have been studied in detail for reproductive biology (Caiola et al., 2000), there is no emphasis on using it as a distinct morphological marker for identifying genuine saffron, once dried or processed.

**Figure 2:**
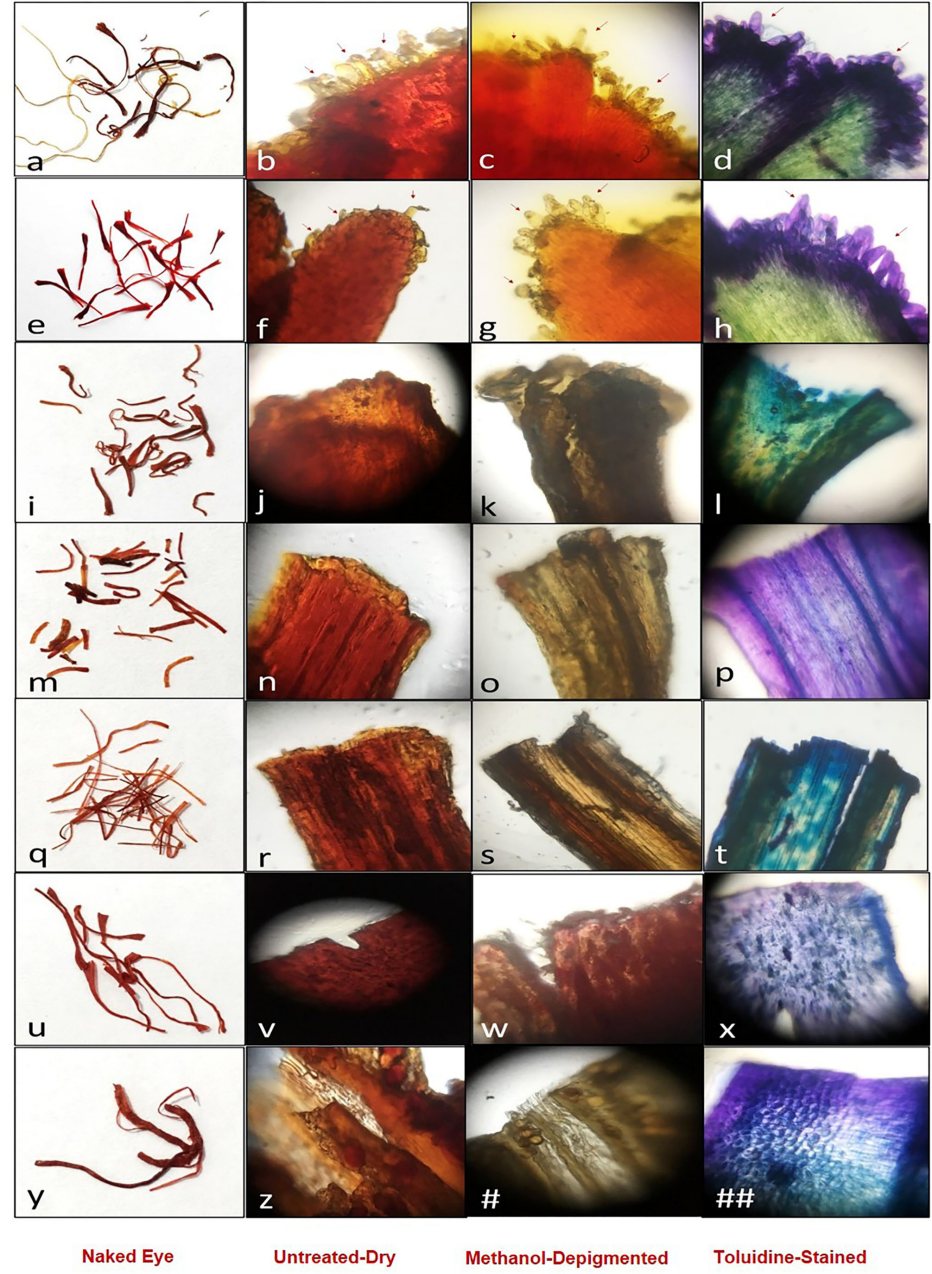
Poster for the identification of genuine/fake saffron: Genuine saffron **(a–d)** saffron in filament (Laccha), **(e–h)** saffron in cut filament (Mongra); fake samples **(i–##)**.

In the present study, we first visualized the filaments of sample classes under a stereo-microscope for a wider field of vision. We observed some differences among the filaments, though these were more conspicuous toward their apices (Supplementary Figure S2). We used Foldscope to focus on the apex area and could easily identify papillae in both commercially available genuine saffron sample classes 1 & 2 (Supplementary Figure S3). Our results show that it is possible to distinguish genuine saffron from its commonly used adulterants or fake look-alikes by detecting the presence of distinct papilla on their trumpet-shaped upper rim (Figures 2b–d,f–h).

While the sample classes we studied showed textural differences between real saffron and the fake ones, serrated top margins and/or pollen grains were seen in authentic as well as spurious sample classes (3, 8) (Supplementary Figure S1). Therefore, unlike the common belief of saffron vendors, the presence of pollen is not a distinctive feature of genuine saffron. Sample class 3 (Supplementary Figure S3c) and sample class 7 (Supplementary Figure S3g) showed serrated top margins, a feature common with authentic saffron; however, both lacked the distinct finger-like projections “papillae.” Similarly, while a large number of pollen grains were seen in sample class 8 and pollen-like granules in sample 3, the papillae were absent in both. Sample classes 4, 5, and 6 (Supplementary Figures S3d–f) featured smooth margins with no serrations, papillae, or pollen grains. Sample class 9 showed smooth, wide top margins with narrower stalk, but lacked the characteristic papillae. Pollen grains were also absent. Overall, sample classes 1, 2, 3, 7, and 9 closely resembled trumpet/funnel-like structure, typical of most stigma, while the remaining four sample classes showed flattened top and margins. The results are summarized in Table 1.

### Creating a poster by de-pigmentation and differential staining

Microscopy is generally used in combination with staining, particularly by school children. Most dyes stain tissues with differing intensities of the same color. However, certain basic dyes stain tissue components with colors other than that of the dye. Such a staining reaction is called metachromasy and is highly selective. Only certain tissue structures stain metachromatically and are said to exhibit metachromasia (Culling et al., 2014).

In the present study, we used staining to further expand the inventory of distinct visual color markers. Toluidine blue (also known as tolonium chloride, methylaniline, or aminotoluene) is used to specifically stain certain components of mucosal lesions and tissue sections owing to its metachromatic property and was first applied for *in vivo* staining of uterine cervical carcinoma *in situ* by Reichart in 1963 (Siddiqui et al., 2006). Inspired by this, we used it to stain filaments, which incidentally showed a distinct differential staining pattern in the case of “genuine” saffron. It stained the papillae and the margins deep purple, while it is lighter yellowish green toward the central axis (Figure 2).

Moreover, when we try to remove the color of filaments, the de-pigmentation of genuine saffron requires more extensive washing than the samples which had been artificially dyed (Figures 2c,g,k,o,s,w,#). Genuine saffron retains most of the color despite washing 3–4-times. These significant findings were used to create a poster showing all the major visual features that a person can use for the manual validation of a given sample (Figure 2).

### Toluidine blue imparts differential stain only to saffron stigma and not fake look-alikes

The staining of samples with toluidine blue in the present study clearly shows the distinct staining pattern based on the histology of papillae (Figure 2). Toluidine blue is partially soluble in both water and alcohol, and selectively stains acidic tissue components like sulfates, carboxylates, and phosphate radicals (Epstein et al., 1992; Gandolfo et al., 2006). Because of these properties, the differences in color intensity of the papillae, their base, and the tissue toward the central axis of saffron stigma are well depicted in toluidine blue staining.

It is known that saffron papillae possess a thick cell wall, covered with a continuous cuticle under which electron-dense material is visible. The papillae contain a large central vacuole, a scarce endoplasmic reticulum, numerous mitochondria and chromoplasts, and virus-like inclusions at the base (Caiola and Chichiriccò, 1991; Caiola et al., 2000). These features are absent in the artificially created fake look-alikes and therefore, get stained uniformly across the whole tissue.

As a metachromatic dye, toluidine absorbs light at different wavelengths, varying with concentration and surroundings, and can change its color without changing its chemical structure. This color change is brought about by the specialized physical changes in the form of stacking of dye cations at regions of the high density of anionic groups in the tissue. Stacking causes a hypsochromic shift (shortens the wavelength of maximum absorption) so that the maximum wavelength of the transmitted light is longer, which makes the observed color look different (Kumar and Kiernan, 2010). The color shift in Figure 2 from a blue or violet dye to a greenish-yellow could represent the polymerization of the dye to varying degrees. The tissue at the margin may have an absorption maximum at 630 nm due to its orthochromatic nature, which, therefore, stains blue. In contrast, the inside tissue stains greenish-yellow, and its absorption spectrum may be closer to 540 nm (Culling et al., 2014). However, these differences are not noticed when the samples are stained with safranin O/iodine/fast green/crystal violet, and all the other staining agents show more or less uniform staining patterns. Similarly, the artificial dyes used by fraudsters to dye the look-alikes of saffron cannot generate the differential pattern shown by toluidine blue.

**Figure 3:**
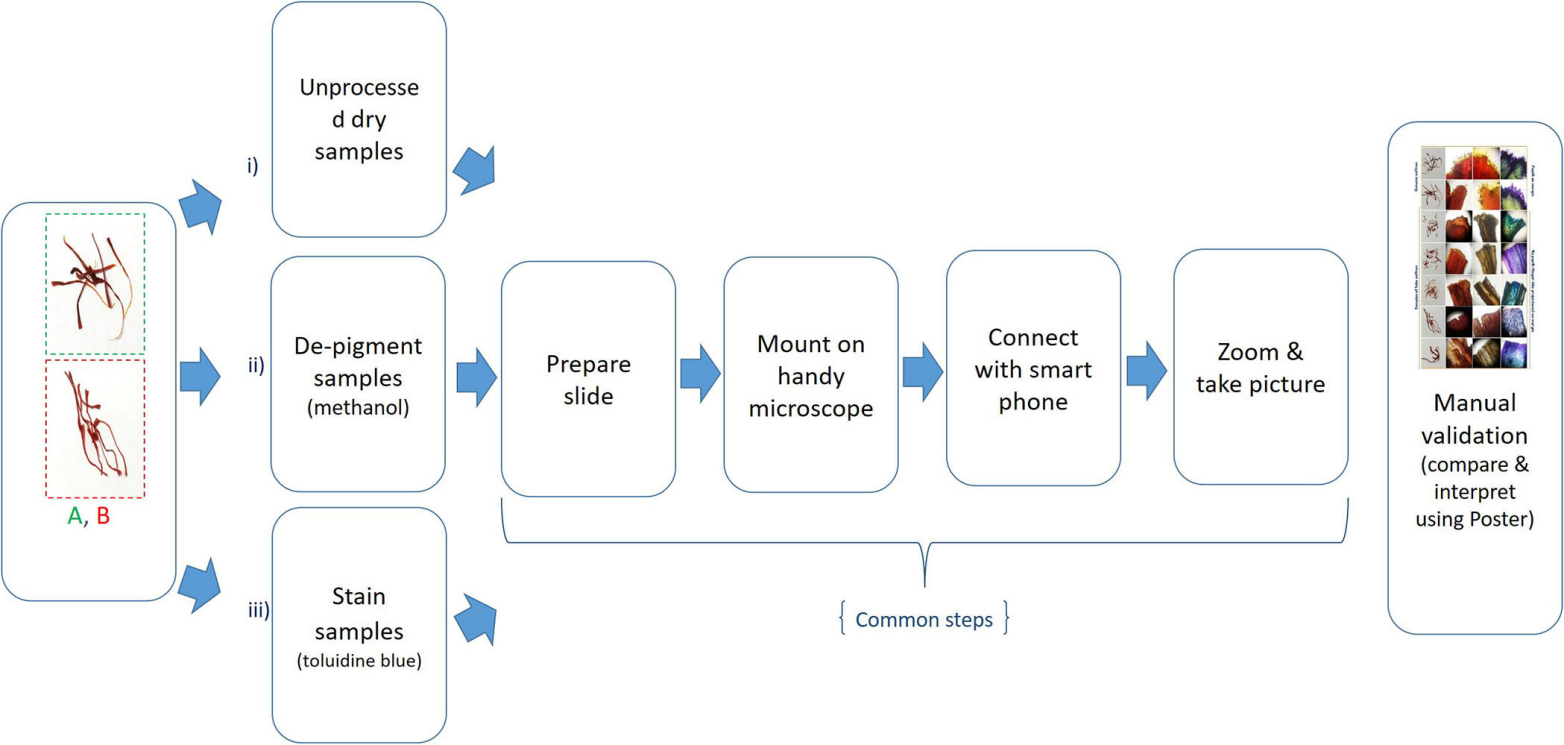
Poster-based validation: (i) Dry samples are used as such (without any chemical treatment) and then common downstream steps, as shown above, are followed; (ii) samples are de-pigmented using methanol first, and then common downstream steps are followed; (iii) samples are stained using toluidine blue, and then common downstream steps are followed.

Based on the above findings, a method was developed for the manual validation of a given sample, and the workflow is shown in Figure 3.

### Machine learning-based approach is quick and robust in detecting fake saffron

Several studies have been conducted on artificial intelligence for identification and classification tasks. However, only a few relevant contributions employ “image classification” in plants. Kurtulmuş et al. (2016) demonstrated using a neural network to classify pepper seed variety based on images. Likewise, Islam et al. (2020) performed flower classification by employing a convolutional neural network on eight different types of flowers and achieved 85% accuracy. The approaches mentioned above focused on identifying the different variety of spices and flowers, while in the present study, we explored end-to-end neural network learning to distinguish the genuine saffron from the fake using image of the sample.

Even when the presence/absence of papillae in genuine/fake saffron is distinct, the approach based on the Canny edge detector multi-stage image processing algorithm did not perform well (Rong et al., 2014). This is due to many factors, like using only gray images (which is needed for edge detection and finding contours) and throwing a lot of information about color schema, texture, etc., and uncertainty in deciding the threshold value of contours, contours created by other structures other than papillae. Moreover, in the machine learning-based approach, we did not process images, and the model learned from all the features available in the sample images, which allowed us to get robustness in performance and much more accurate results (Janiesch et al., 2021; Greener et al., 2022). We compared all the ML-based saffron classification models on validation and test datasets (Table 2). We observed that the deep neural networks (ResNet18 and DenseNet121) performed better than random forest (RF) and support vector machine (SVM)-based approaches (Kremic and Subasi, 2016; Speiser et al., 2019; Nandhini and Ashokkumar, 2022; Zhou et al., 2022). Also, the best models (two in total) out of the three variations per deep neural network are the models using the pre-trained model weights and freezing all the layers, apart from the last two layers. Both the models recorded 99.5% accuracy with a precision of 99.3% on the test dataset, which is pretty decent. The models show 99.5% accuracy and precision of 99.1% on the validation dataset. This shows that the models are generalized and behave almost the same on the validation and the test dataset. Further, this approach has accelerated the decision-making process regarding the genuineness of a sample image as it takes less than a second per photograph.

**Table 2:** Quantitative comparison of ML-based classification models for saffron classification based on accuracy and precision.

**Model**	**Training precision**	**Training accuracy**	**Validation precision**	**Validation accuracy**	**Test precision**	**Test accuracy**
Random Forest	1.0	1.0	0.94	0.94	0.95	0.95
SVC	0.95	0.95	0.90	0.90	0.95	0.95
Resnet18 with random weight initialization	0.96	0.93	0.91	0.91	0.93	0.92
Resnet18 Pretrained—unfreeze	0.97	0.96	0.93	0.92	0.95	0.93
Resnet18 pretrained	0.99	0.99	0.99	0.99	0.99	0.99
Densenet121 with random weight initialization	0.96	0.95	0.95	0.93	0.97	0.93
Densenet121 Pretrained—unfreeze	0.95	0.94	0.91	0.89	0.94	0.90
Densenet121 pretrained	0.99	0.99	0.99	0.99	0.99	0.99

**Figure 4:**
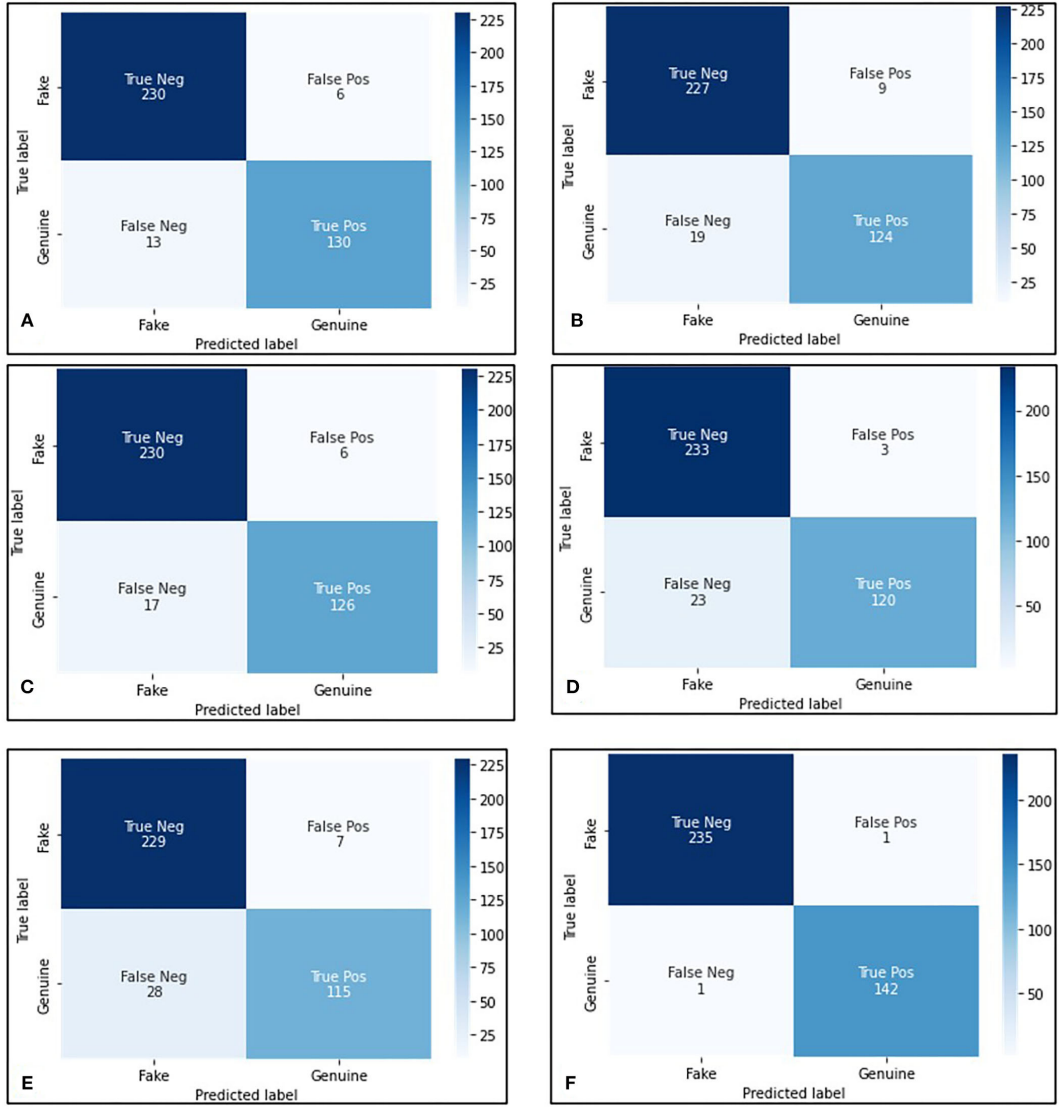
Quantitative analysis based on the test dataset. Confusion Matrix for: **(A)** random forestand SVM, **(B)** ResNet18 with random weight initialization, **(C)** ResNet18 pre-trained—network is not in freeze state, **(D)** DenseNet121 with random weight initialization, **(E)** DenseNet121 pre-trained—network is not in freeze state, and **(F)** ReNet18 and DenseNet121 pre-trained network, with all the layers in freeze state, but the last two layers.

Figure 4 illustrates the contrastive results based on the confusion matrix. We observed that the deep neural network-based approaches (ResNet18 and DenseNet121 with pre-trained model weights and freezing all the layers, apart from the last two layers) demonstrate maximal performance with 235 fake samples correctly detected out of 236, and 142 rightly predicted as genuine out of 143 genuine saffron samples on the test dataset. Only one sample from fake and genuine was wrongly classified, as highlighted (Figure 4).

**Figure 5:**
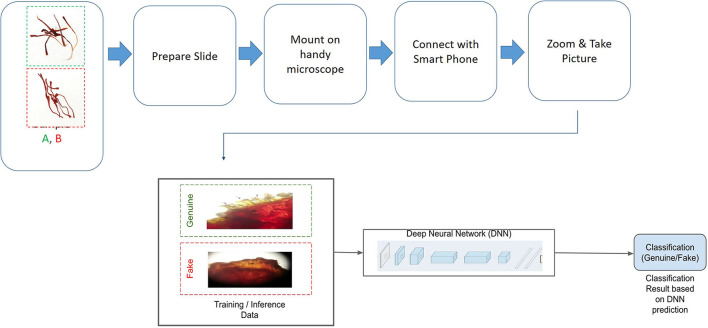
App-based detection: Deep neural network is trained on a dataset comprising genuine and fake saffron samples. Trained model is used to generate inference based on samples to predict the classification, i.e., whether the sample is genuine or fake.

The machine learning-based classification approach automated and simplified the process to make detecting fake/genuine saffron quicker. The workflow for the process is shown in Figure 5.

### Adulteration and quality estimation

**Figure 6:**
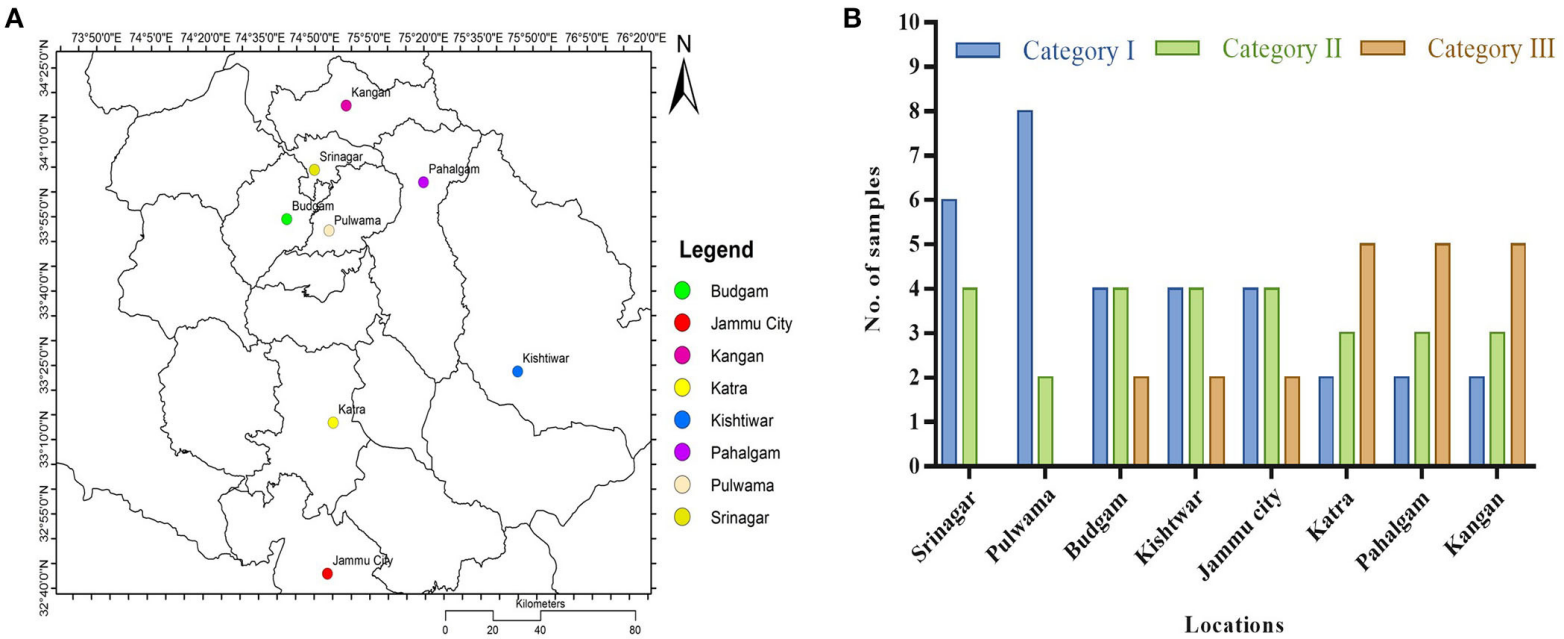
Saffron market-sample collection sites for assessing adulteration and quality: **(A)** Map depicting geographical locations; **(B)** categorization of the 80 samples according to ISO 3632 standard.

Eighty market samples (1 g each) were used to assess adulteration and quality. The geographical coordinates and the locations of the eight cities/towns from where these samples were procured are shown using the ESRI ArcGIS map (Figure 6A). The names of the sites and the localities are: (1) Budgam (Chadoora, Budgam, Magam, Beerwah, Humhama), (2) Jammu city (Trikuta Nagar, Gandhi Nagar, Raghunath Bazar, Janipur, Chani Himat), (3) Kangan (Kangan town, Dursuma, Wussan, Preng, Cherwan), (4) Katra (Katra town, Dhar Vaishno Devi, Akhli, Bhangal, Arli, Hansali), (5) Kishtwar (Poochal, Matta, Janwas, Dool, Ohli), (6) Pahalgam (Pahalgam town, Ashmukam, Salar, Dirhama, Batkoot), (7) Pulwama (Lethpora, Pampore, Awantipora, Namlabal, Konibal), and (8) Srinagar (Lalchowk, Dalgate, Sonwar, Dargah, Nowhatta).

Out of a total of 80 market samples, the number of adulterated samples was 48 (Figure 6B). Twenty samples from Pulwama and Srinagar showed a cumulative average adulteration of <1.5%, while the 30 samples from Budgam, Kishtwar, and Jammu city showed around 16%. The samples (30) from Katra, Pahalgam, and Kangan were highly adulterated, with an average of 40% adulteration. The saffron bought from Pulwama and Srinagar showed adulteration between 0.12 and 2.36% (among the 20 samples), and for the quality, they fall into Categories I and II of ISO 3632 (1 & 2) standards (Figure 6B).

The overall percentage of saffron samples which belonged to Categories II and III is 60% (Figure 6B). While the adulteration among these adulterated samples ranged from 2.09 to 71.23%, and their mean adulteration percentage was 36.25%. Surprisingly, adulteration strongly correlated with the location of sample procurement. Srinagar and Pulwama showed minimal adulteration problems, perhaps because of stricter law enforcement agencies or more awareness among the sellers and buyers. This location-dependent adulteration shows that fraudsters know that cheating would go undetected at places where demand is more due to the tourist rush while its supply is limited.

**Figure 7:**
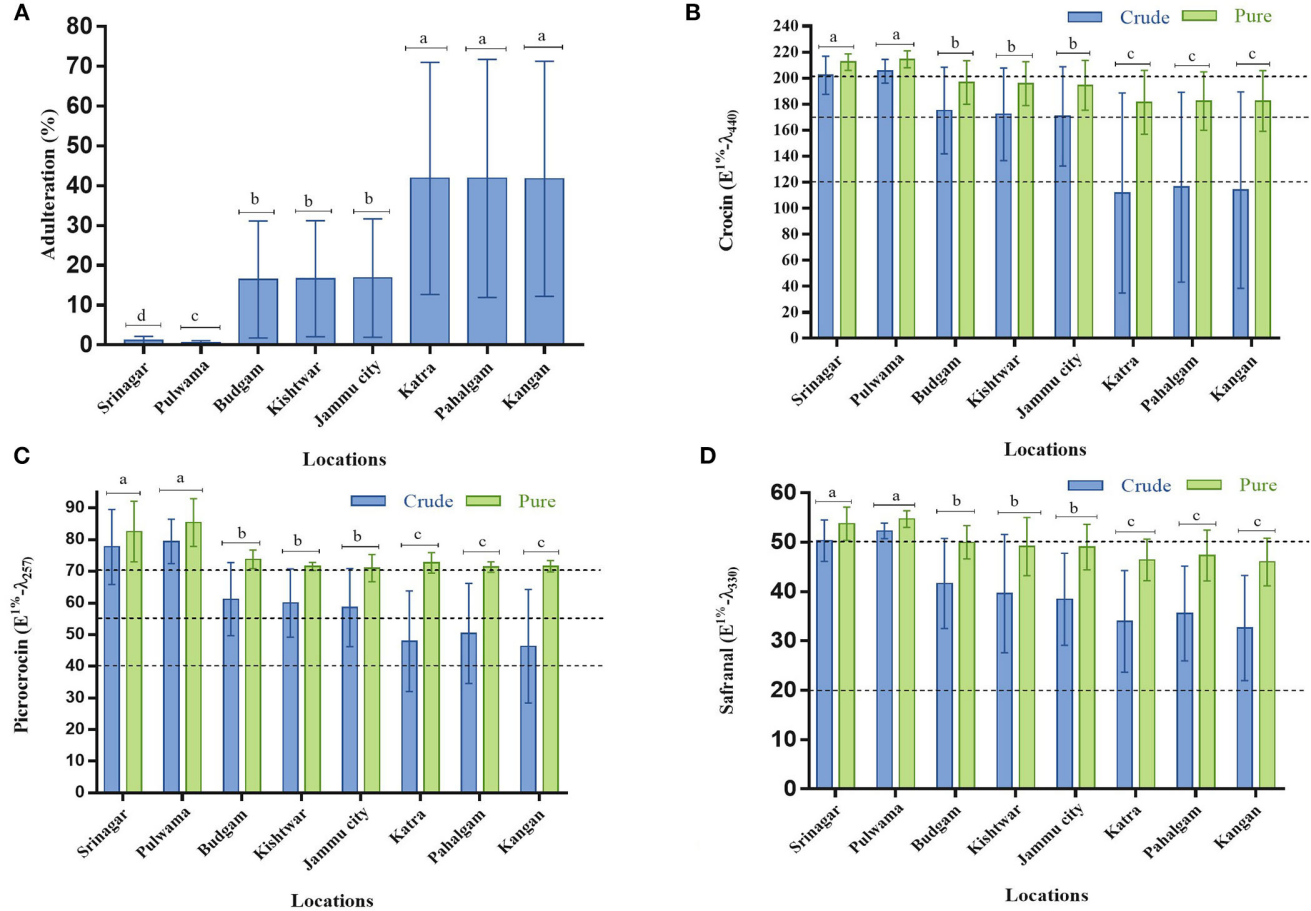
Assessment of saffron quality obtained from different locations of Jammu and Kashmir (India) in pure and adulterated samples: **(A)** Adulteration percent, **(B)** crocin, **(C)** picrocrocin, and **(D)** safranal content. The dotted lines represent the maximum and minimum values of crocin, picrocrocin, and safranal for the categorization of saffron quality as per ISO 3632 standards. Values are the mean of 10 replicates and expressed as mean ± S.D (standard deviation). The letters a, b and c indicate a statistically significant difference at *p* ≤ 0.05 probability level between different locations. Bars with no common letters are significantly different (*p* ≤ 0.05).

While the saffron quality depends on many factors (Husaini, 2014), a significant reduction in the quality of color (crocin), bitterness (picrocrocin), and aroma (safranal) was recorded in the adulterated samples which are inversely proportional to the adulteration percentage (Figure 7). After removing the adulterant from the crude market sample, the sample quality improved significantly, pushing some from Category III to Category II (Figure 7B). It infers that Categories II and III saffron are more likely to be adulterated by fraudsters (Figures 6B, 7A).

### Practical applications

Saffron is a costly spice used as a routine in common people's religious rituals and local cuisines (Husaini and Wani, 2020). However, it is evident from the above data that saffron adulteration and fraud are a big menace. People prefer to buy it as “filaments” because it is easier to use from the dosage point of view, and there are lesser chances of adulteration than in the powdered form. However, unfortunately, some fraudsters have even found ways to “create” fake saffron-like filaments.

Saffron dealers can use the two methods developed in the present study (Figures 3, 6) to showcase the authenticity of saffron to their customers without much botheration. The customer can himself check the adulteration percentage in a random sample and identify fake saffron by using a simple application on a mobile phone. It would act as an additional check for the fraudsters who manage to get fake GI tags, holograms, and certifications and sell their products in the unorganized sector while going undetected by the law enforcement agencies (Husaini et al., 2010a). Further, genuine retailers can convince the customers about the authenticity of their products by showing these visual markers using Foldscope or by installing the mobile app on their smartphone, thereby promoting genuine retail business.

Furthermore, we created a kit for commercial use and quality control laboratories (the patent is under process). The kit constitutes a poster showing distinct and unique markers in saffron stigma, a Foldscope, slides, cello-tape, methanol, and toluidine solution. These methods have the potential to be put to use in the European Science Foundation-sponsored COST Action FA1101 (Saffron-omics: Omics technologies for crop improvement, traceability, determination of authenticity, adulteration, and origin). The results of the de-pigmentation and the staining procedures can be used to update the relevant sections of “test methods” in the identification test and microscopic examination of the International Standards Organization ISO 3632 (1 & 2).

## Conclusion

Individual consumers prefer to buy “saffron in filaments” (Laccha), or “saffron in cut filaments” (Mongra) because it is easier to use from the dosage point of view, and there are lesser chances of adulteration than in the powdered form. However, fraudsters have invented fake saffron-like filaments to cheat customers. We developed two Foldscope-based techniques for the identification of pure saffron. One technique uses a simple visual comparison of distinct markers (papillae of stigma) with a poster, and the other uses an automated approach through a mobile application. Machine learning simplifies the process and automates the detection of fake/genuine saffron samples. It enables end-users not to worry about identifying samples from magnified images themselves. This approach accelerates the whole identification process and takes less than a second per sample after acquiring its image. While large-scale testing of saffron quality using sophisticated methods in specialized laboratories shall always be required for the saffron industry, we have added a new dimension by bringing the customer to the forefront.

Adulteration remains a significant challenge to the saffron industry. The quality of a saffron sample decreases significantly with an increase in adulteration percentage. A critical observation of the present study is that the ISO Category I saffron is not subjected to adulteration, perhaps because elite customers are willing to pay higher prices and generally buy from trusted sources. Saffron belonging to the ISO Categories II and III is more prone to adulteration as it is available freely everywhere as innocent customers prefer to buy saffron at cheaper rates from untrusted sources!

## Data availability statement

The raw data supporting the conclusions of this article will be made available by the authors, without undue reservation.

## Author contributions

AH conceived the idea, designed, directed, performed, and coordinated the research experiments with the active participation of SH, AS, and AW. MD planned and performed machine learning to analyze and automate the process. AH and MD wrote the paper together. All authors contributed to the article and approved the submitted version.

## Conflict of interest

The authors declare that the research was conducted in the absence of any commercial or financial relationships that could be construed as a potential conflict of interest.

## Publisher's note

All claims expressed in this article are solely those of the authors and do not necessarily represent those of their affiliated organizations, or those of the publisher, the editors and the reviewers. Any product that may be evaluated in this article, or claim that may be made by its manufacturer, is not guaranteed or endorsed by the publisher.

## Supplementary material

Supplementary Figure 1 Pollen on **(a)** freshly cut saffron stigma; **(b)** sample class 1; **(c)** sample class 2; **(d)** sample class 3; **(e)** sample class 8; **(f)** saffron stigma mounted on a slide in Foldscope.

Supplementary Figure 2 Stereomicroscopic images of the apices of filament sample classes at a magnification of 80× **(a–i)**.

Supplementary Figure 3 Foldscope images of top region/margin of **(a)** sample class 1 showing papillae; **(b)** sample class 2 showing papillae; **(c)** sample class 3 showing serrations; **(d)** sample class 4 showing smooth margin with no serrations; **(e)** sample class 5 showing even margin without serrations; **(f)** sample class 6 showing smooth edges without serrations; **(g)** sample class 7; **(h)** sample class 8; **(i)** sample class 9 showing smooth surface.
