# Supplementary figures and images for: The menace of saffron adulteration: Low-cost rapid identification of fake look-alike saffron using Foldscope and machine learning technology
Source: Front Plant Sci. 2022 Aug 12;13:945291. doi: 10.3389/fpls.2022.945291 (PMC9417335; doi:10.3389/fpls.2022.945291)

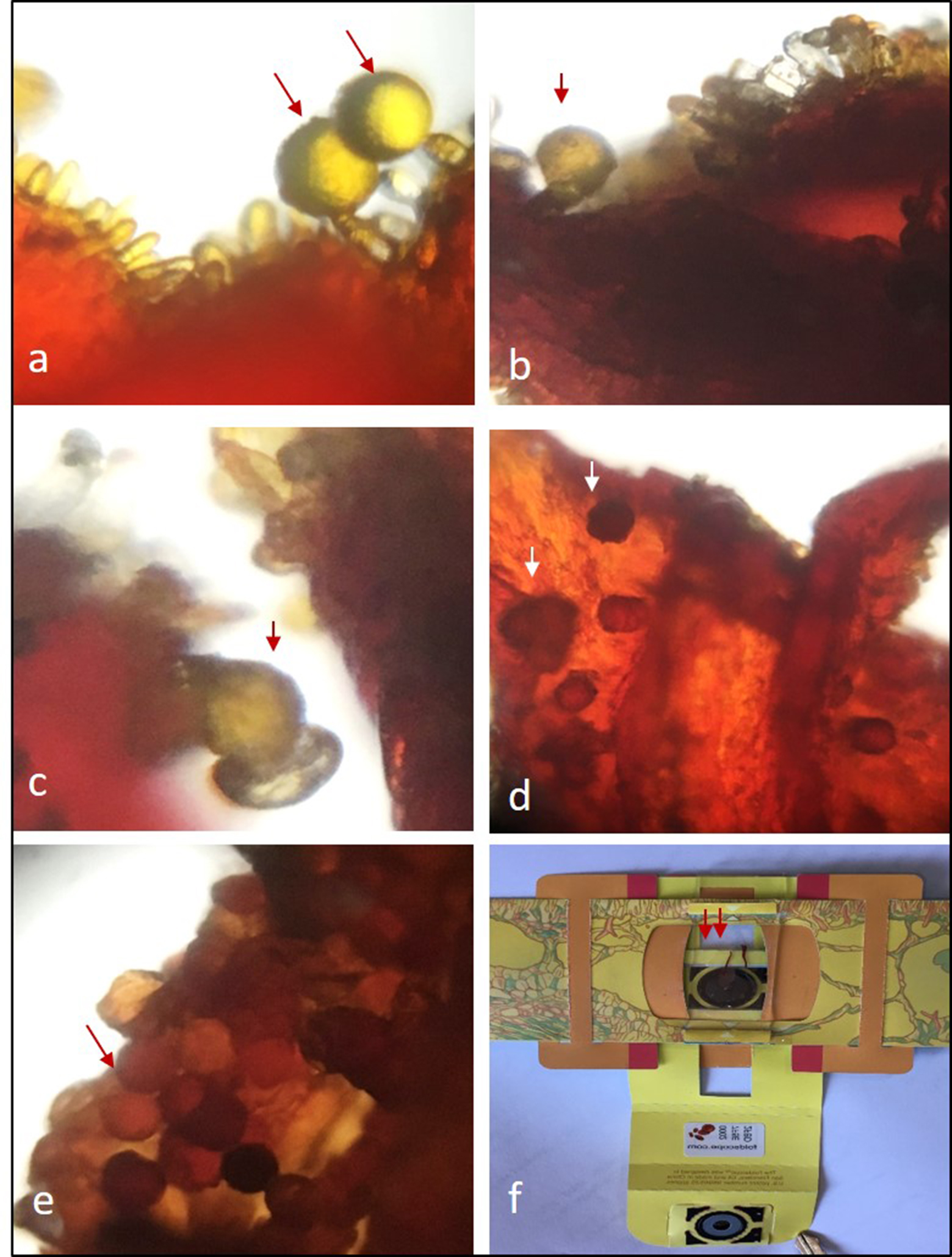

Supplement: Supplementary Figure 1 — Pollen on (a) freshly cut saffron stigma; (b) sample class 1; (c) sample class 2; (d) sample class 3; (e) sample class 8; (f) saffron stigma mounted on a slide in Foldscope. [file Image_1.JPEG]

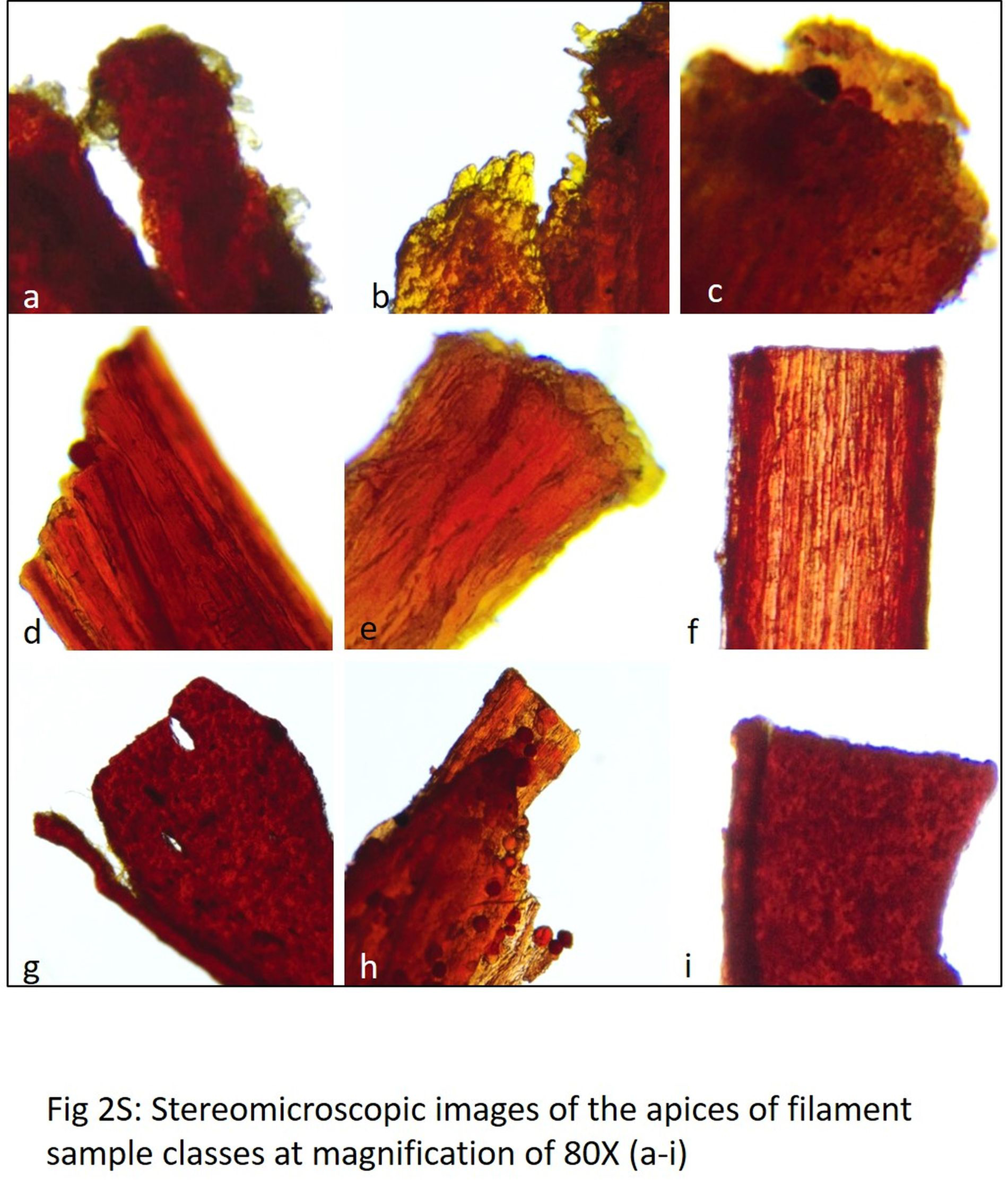

Supplement: Supplementary Figure 2 — Stereomicroscopic images of the apices of filament sample classes at a magnification of 80× (a–i). [file Image_2.jpeg]

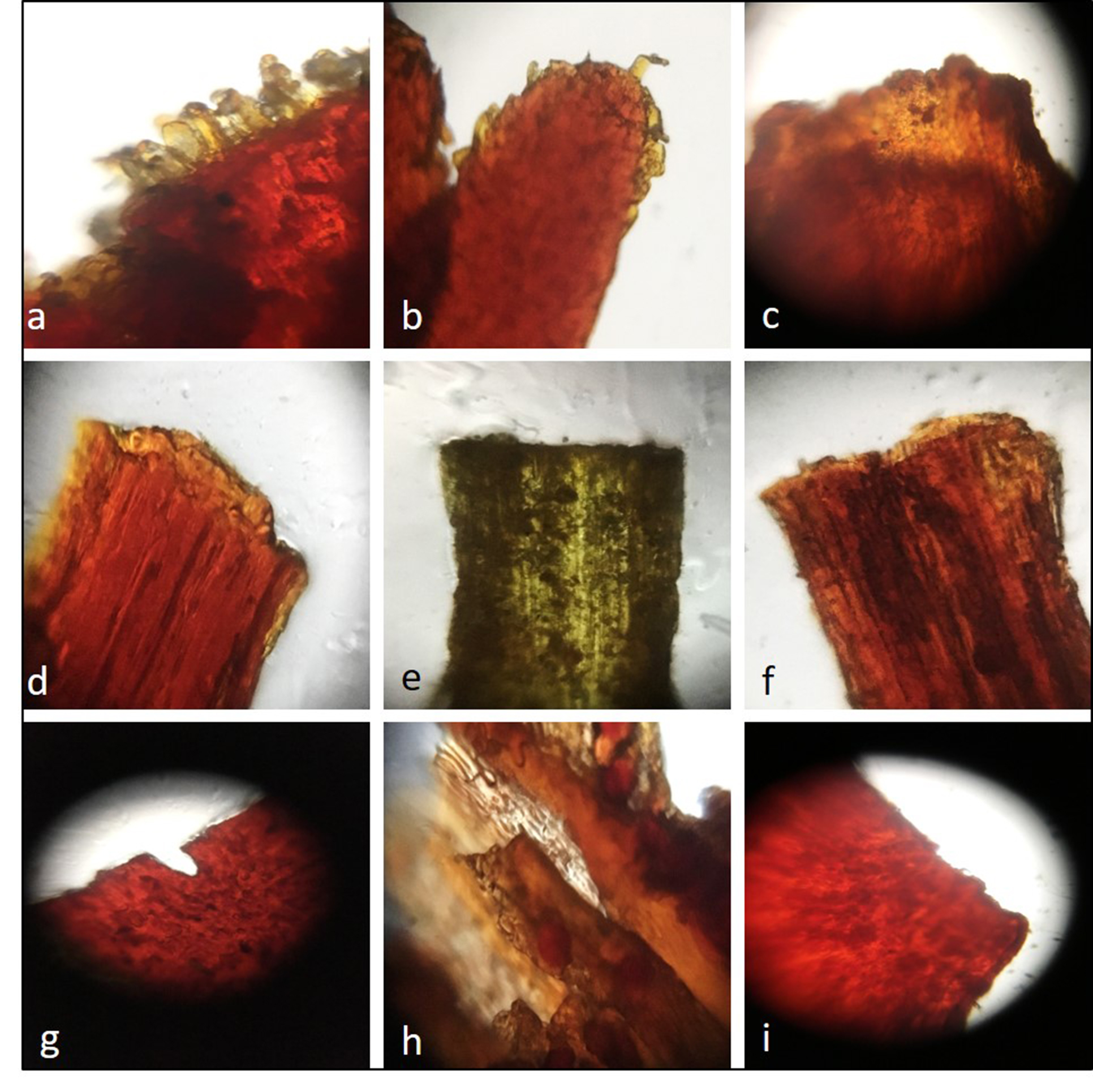

Supplement: Supplementary Figure 3 — Foldscope images of top region/margin of (a) sample class 1 showing papillae; (b) sample class 2 showing papillae; (c) sample class 3 showing serrations; (d) sample class 4 showing smooth margin with no serrations; (e) sample class 5 showing even margin without serrations; (f) sample class 6 showing smooth edges without serrations; (g) sample class 7; (h) sample class 8; (i) sample class 9 showing smooth surface. [file Image_3.jpeg]
